# Depression prevalence based on the Edinburgh Postnatal Depression Scale compared to Structured Clinical Interview for DSM DIsorders classification: Systematic review and individual participant data meta‐analysis

**DOI:** 10.1002/mpr.1860

**Published:** 2020-10-22

**Authors:** Anita Lyubenova, Dipika Neupane, Brooke Levis, Yin Wu, Ying Sun, Chen He, Ankur Krishnan, Parash M. Bhandari, Zelalem Negeri, Mahrukh Imran, Danielle B. Rice, Marleine Azar, Matthew J. Chiovitti, Nazanin Saadat, Kira E. Riehm, Jill T. Boruff, John P. A. Ioannidis, Pim Cuijpers, Simon Gilbody, Lorie A. Kloda, Scott B. Patten, Ian Shrier, Roy C. Ziegelstein, Liane Comeau, Nicholas D. Mitchell, Marcello Tonelli, Simone N. Vigod, Franca Aceti, Jacqueline Barnes, Amar D. Bavle, Cheryl T. Beck, Carola Bindt, Philip M. Boyce, Adomas Bunevicius, Linda H. Chaudron, Nicolas Favez, Barbara Figueiredo, Lluïsa Garcia‐Esteve, Lisa Giardinelli, Nadine Helle, Louise M. Howard, Jane Kohlhoff, Laima Kusminskas, Zoltán Kozinszky, Lorenzo Lelli, Angeliki A. Leonardou, Valentina Meuti, Sandra N. Radoš, Purificación N. García, Susan J. Pawlby, Chantal Quispel, Emma Robertson‐Blackmore, Tamsen J. Rochat, Deborah J. Sharp, Bonnie W. M. Siu, Alan Stein, Robert C. Stewart, Meri Tadinac, S. Darius Tandon, Iva Tendais, Annamária Töreki, Anna Torres‐Giménez, Thach D. Tran, Kylee Trevillion, Katherine Turner, Johann M. Vega‐Dienstmaier, Andrea Benedetti, Brett D. Thombs

**Affiliations:** ^1^ Lady Davis Institute for Medical Research Jewish General Hospital Montréal Québec Canada; ^2^ Department of Epidemiology Biostatistics and Occupational Health McGill University Montréal Québec Canada; ^3^ School of Primary Centre for Prognosis Research Community and Social Care Keele University Keele Staffordshire UK; ^4^ Department of Psychiatry McGill University Montréal Québec Canada; ^5^ Department of Psychology McGill University Montréal Québec Canada; ^6^ Department of Mental Health Bloomberg School of Public Health Johns Hopkins University Baltimore Maryland USA; ^7^ Schulich Library of Physical Sciences, Life Sciences, and Engineering McGill University Montréal Québec Canada; ^8^ Department of Medicine Stanford University Stanford California USA; ^9^ Department of Health Research and Policy Stanford University Stanford California USA; ^10^ Department of Biomedical Data Science Stanford University Stanford California USA; ^11^ Department of Statistics Stanford University Stanford California USA; ^12^ Department of Clinical, Neuro and Developmental Psychology EMGO Institute Vrije Universiteit Amsterdam Amsterdam the Netherlands; ^13^ Hull York Medical School and the Department of Health Sciences University of York Heslington York UK; ^14^ Library Concordia University Montréal Québec Canada; ^15^ Departments of Community Health Sciences and Psychiatry University of Calgary Calgary Alberta Canada; ^16^ Mathison Centre for Mental Health Research & Education University of Calgary Calgary Alberta Canada; ^17^ Cuthbertson & Fischer Chair in Pediatric Mental Health University of Calgary Calgary Alberta Canada; ^18^ Department of Family Medicine McGill University Montréal Québec Canada; ^19^ Department of Medicine Johns Hopkins University School of Medicine Baltimore Maryland USA; ^20^ International Union for Health Promotion and Health Education École de santé publique de l'Université de Montréal Montréal Québec Canada; ^21^ Department of Psychiatry University of Alberta Edmonton Alberta Canada; ^22^ Alberta Health Services Edmonton Alberta Canada; ^23^ Department of Medicine University of Calgary Calgary Alberta Canada; ^24^ Women's College Hospital and Research Institute University of Toronto Toronto Ontario Canada; ^25^ Department of Neurology and Psychiatry Sapienza University of Rome Rome Italy; ^26^ Department of Psychological Sciences Birkbeck, University of London London UK; ^27^ Department of Psychiatry Rajarajeswari Medical College and Hospital Bengaluru Karnataka India; ^28^ University of Connecticut School of Nursing Mansfield Connecticut USA; ^29^ Department of Child and Adolescent Psychiatry University Medical Center Hamburg‐Eppendorf Hamburg Germany; ^30^ Discipline of Psychiatry Westmead Clinical School Sydney Medical School University of Sydney Sydney New South Wales Australia; ^31^ Department of Psychiatry Westmead Hospital Sydney New South Wales Australia; ^32^ Neuroscience Institute Lithuanian University of Health Sciences Kaunas Lithuania; ^33^ Departments of Psychiatry Pediatrics Obstetrics and Gynecology School of Medicine and Dentistry University of Rochester Rochester New York USA; ^34^ Faculty of Psychology and Educational Sciences University of Geneva Geneva Switzerland; ^35^ IUP University of Lausanne Lausanne Switzerland; ^36^ School of Psychology University of Minho Braga Portugal; ^37^ Perinatal Mental Health Unit CLINIC‐BCN Institut Clínic de Neurociències Hospital Clínic Barcelona Spain; ^38^ Vulnerability, Psychopathology and Gender Research Group Barcelona Spain; ^39^ August Pi i Sunyer Biomedical Research Institute Barcelona Spain; ^40^ Department of Health Sciences Psychiatry Unit University of Florence Firenze Italy; ^41^ Institute of Psychiatry, Psychology & Neuroscience King's College London London UK; ^42^ South London and Maudsley NHS Foundation Trust London UK; ^43^ School of Psychiatry University of New South Wales Kensington New South Wales Australia; ^44^ Ingham Institute Liverpool New South Wales Australia; ^45^ Karitane Carramar New South Wales Australia; ^46^ Private Practice Hamburg Germany; ^47^ Department of Obstetrics and Gynecology Danderyd Hospital Stockholm Sweden; ^48^ First Department of Psychiatry Women's Mental Health Clinic Athens University Medical School Athens Greece; ^49^ Department of Psychology Catholic University of Croatia Zagreb Croatia; ^50^ Psychology Service Regidoria de Polítiques de Gènere Ajuntament de Terrassa Terrassa Spain; ^51^ Department of Obstetrics and Gynaecology Albert Schweitzer Ziekenhuis Dordrecht the Netherlands; ^52^ Halifax Health Graduate Medical Education Daytona Beach Florida USA; ^53^ MRC/Developmental Pathways to Health Research Unit School of Clinical Medicine University of Witwatersrand Johannesburg South Africa; ^54^ Human and Social Development Programme Human Sciences Research Council Pretoria South Africa; ^55^ Centre for Academic Primary Care Bristol Medical School University of Bristol Bristol UK; ^56^ Department of Psychiatry Castle Peak Hospital Hong Kong SAR China; ^57^ Department of Psychiatry University of Oxford Oxford UK; ^58^ MRC/Wits Rural Public Health and Health Transitions Research Unit School of Public Health Faculty of Health Sciences University of the Witwatersrand Johannesburg South Africa; ^59^ Division of Psychiatry University of Edinburgh Edinburgh Scotland UK; ^60^ Malawi Epidemiology and Intervention Research Unit (MEIRU) Lilongwe Malawi; ^61^ Department of Psychology Faculty of Humanities and Social Sciences University of Zagreb Zagreb Croatia; ^62^ Feinberg School of Medicine Northwestern University Chicago Illinois USA; ^63^ Department of Emergency University of Szeged Szeged Hungary; ^64^ School of Public Health and Preventive Medicine Monash University Melbourne Victoria Australia; ^65^ Epilepsy Center‐Child Neuropsychiatry Unit ASST Santi Paolo Carlo San Paolo Hospital Milan Italy; ^66^ Facultad de Medicina Alberto Hurtado Universidad Peruana Cayetano Heredia Lima Perú; ^67^ Respiratory Epidemiology and Clinical Research Unit McGill University Health Centre Montréal Québec Canada; ^68^ Department of Medicine McGill University Montréal Québec Canada; ^69^ Department of Educational and Counselling Psychology McGill University Montréal Québec Canada; ^70^ Biomedical Ethics Unit McGill University Montréal Québec Canada

**Keywords:** depression prevalence, Edinburgh Postnatal Depression Scale, structured clinical interview for DSM, individual participant data meta‐analysis, major depression

## Abstract

**Objectives:**

Estimates of depression prevalence in pregnancy and postpartum are based on the Edinburgh Postnatal Depression Scale (EPDS) more than on any other method. We aimed to determine if any EPDS cutoff can accurately and consistently estimate depression prevalence in individual studies.

**Methods:**

We analyzed datasets that compared EPDS scores to Structured Clinical Interview for DSM (SCID) major depression status. Random‐effects meta‐analysis was used to compare prevalence with EPDS cutoffs versus the SCID.

**Results:**

Seven thousand three hundred and fifteen participants (1017 SCID major depression) from 29 primary studies were included. For EPDS cutoffs used to estimate prevalence in recent studies (≥9 to ≥14), pooled prevalence estimates ranged from 27.8% (95% CI: 22.0%–34.5%) for EPDS ≥ 9 to 9.0% (95% CI: 6.8%–11.9%) for EPDS ≥ 14; pooled SCID major depression prevalence was 9.0% (95% CI: 6.5%–12.3%). EPDS ≥14 provided pooled prevalence closest to SCID‐based prevalence but differed from SCID prevalence in individual studies by a mean absolute difference of 5.1% (95% prediction interval: −13.7%, 12.3%).

**Conclusion:**

EPDS ≥14 approximated SCID‐based prevalence overall, but considerable heterogeneity in individual studies is a barrier to using it for prevalence estimation.

## 1 INTRODUCTION

Accurate estimates of depression prevalence are necessary to understand disease burden and allocate healthcare resources. Validated diagnostic interviews, such as the Composite International Diagnostic Interview (Wittchen, 1994) and the Structured Clinical Interview for the DSM (SCID) (First & Gibbon, 2004) are designed to classify major depression and estimate depression prevalence in a manner consistent with diagnostic criteria. However, administering validated diagnostic interviews to samples that are large enough to estimate prevalence is resource‐intensive. Thus, many researchers administer self‐report depression symptom questionnaires, or screening tools, instead, and report the percentage above a cutoff threshold as the prevalence of depression (Levis et al., 2019b; Thombs, Kwakkenbos, Levis, & Benedetti, 2018).

Some items included in self‐report questionnaires address similar symptoms as those evaluated in validated diagnostic interviews, but most questionnaires do not evaluate all relevant symptoms, and most include other items that are not part of diagnostic criteria. Furthermore, unlike validated diagnostic interviews, self‐report questionnaires do not include historical information necessary for differential diagnosis, investigate non‐psychiatric medical conditions that can cause similar symptoms to those of depression, assess functional impairment related to symptoms, or verify that symptoms are not an expected reaction to losses or stressors (Thombs et al., 2018).

Depression screening tools are designed to cast a wide net and identify individuals who may have depression. Individuals who screen positive on depression screening tools must be further evaluated by a trained health care professional to confirm whether diagnostic criteria are met. Based on sensitivity and specificity estimates for common depression screening tools and cutoff thresholds, if depression screening tools are used to attempt to estimate prevalence rather than identify individuals who may have depression, most would be expected to overestimate prevalence compared to actual diagnoses (Levis et al., 2019b; Thombs et al., 2018).

A recent study that examined 69 meta‐analyses of depression prevalence found that 44% of pooled prevalence estimates in meta‐analysis abstracts were based solely on screening or rating tools and 46% on a combination of screening tools and other methods (e.g., unstructured interviews, medical charts); only 10% were based solely on diagnostic interviews (Levis et al., 2019b). Among 2094 primary studies included in the meta‐analyses, 77% used screening or rating tools, whereas only 13% used validated diagnostic interviews exclusively. Meta‐analyses based solely on screening or rating tools reported an average depression prevalence of 31% compared to 17% in meta‐analyses based solely on diagnostic interviews.

The degree to which screening questionnaires overestimate the true prevalence depends on the specific depression screening tool and cutoff threshold used (Levis et al., 2019b; Thombs et al., 2018). To date, we are aware of only one study that has directly compared prevalence based on a specific screening tool and cutoff threshold to prevalence based on a validated diagnostic interview for major depression (Levis et al., 2020). That study, an individual participant data meta‐analysis (IPDMA), included 9242 participants from 44 primary studies who were administered both the Patient Health Questionnaire‐9 (PHQ‐9) and the SCID diagnostic interview and found that prevalence based on the standard PHQ‐9 cutoff of ≥10 was 25% (95% confidence interval [CI]: 21%–30%), compared to 12% (95% CI: 10%–15%) based on the SCID. The study also reported that no PHQ‐9 cutoff consistently matched prevalence based on the SCID in individual studies.

The 10‐item Edinburgh Postnatal Depression Scale (EPDS) (Cox, Holden, & Sagovsky, 1987) is the most commonly used depression screening tool for women in pregnancy or postpartum (Hewitt et al., 2009; Howard et al., 2014). It was designed for assessing symptoms continuously, for providing information for discussion for patients, and to identify women who may benefit from formal mental health assessment (Cox et al., 1987). We reviewed 53 recently published studies that stated in their title or abstract that they assessed prevalence of “depression”, “depressive disorders”, “major depression” or “major depressive disorder”. We excluded any that stated that they reported the prevalence of “depressive symptoms” or similar terms. We found that only 6 (11%) used a validated diagnostic interview designed for this purpose. There were 26 (49%) studies that used the EPDS and 21 studies that used other methods, mostly other questionnaires. Studies that reported prevalence based on the EPDS used cutoff thresholds from ≥9 to ≥14, with the majority using cutoffs of ≥10 and ≥ 13 (see Supplementary material, Methods S1 and Table S1). The extent to which prevalence estimates based on different EPDS cutoffs may differ from prevalence based on validated diagnostic interviews, however, is unknown.

The aim of the present study was to use an IPDMA approach to (1) determine the degree to which EPDS cutoffs that are commonly used to report depression prevalence may deviate from prevalence based on a validated semi‐structured diagnostic interview, the SCID; and (2) to use a prevalence matching approach (Kelly, Dunstan, Lloyd, & Fone, 2008; Thombs et al., 2018) to determine whether any cutoff threshold on the EPDS matches SCID major depression prevalence closely and with sufficiently low heterogeneity to be used for estimating major depression prevalence in individual studies.

## 2 METHODS

We used a subset of data accrued for an IPDMA on EPDS diagnostic accuracy. The IPDMA was registered in PROSPERO (CRD42015024785), and a protocol was published (Thombs et al., 2015). The present study was not included in the protocol for the main EPDS IPDMA, but a separate protocol was published on the Open Science Framework prior to initiating the study (https://osf.io/7gy6p/).

### 2.1 Identification of eligible studies

In the main IPDMA, datasets from articles in any language were eligible for inclusion if (1) they included EPDS scores for women who were pregnant or in the postpartum period, defined as within 12 months of birth; (2) they included diagnostic classifications for current Major Depressive Episode or Major Depressive Disorder based on DSM (American Psychiatric Association, 1987, 1994, 2000, 2013) or International Classification of Diseases (World Health Organization, 1992) criteria, using a validated semi‐structured or fully structured interview; (3) the EPDS and diagnostic interview were administered within 2 weeks of each other, since diagnostic criteria for major depression are for symptoms in the last 2 weeks; (4) participants were ≥18 years and not recruited from youth or school‐based settings; and (5) participants were not recruited from psychiatric settings or because they were identified as having symptoms of depression, since screening is done to identify unrecognized cases. Datasets where not all participants were eligible were included if primary data allowed selection of eligible participants.

For the present study, in our main analyses, we included only primary studies that based major depression diagnoses on the SCID (First & Gibbon, 2004). The SCID is a semi‐structured diagnostic interview intended to be conducted by an experienced diagnostician; it requires clinical judgment and allows rephrasing questions and probes to follow up responses. The reason for including only studies that administered the SCID is because semi‐structured interviews replicate diagnostic standards more closely than other types of interviews, and the SCID is by far the most commonly used semi‐structured diagnostic interview for depression research (Levis et al., 2018, 2019a; Wu et al., 2020). In recent analyses using three large IPDMA databases (Levis et al., 2018, 2019a; Wu et al., 2020), compared to semi‐structured interviews, fully structured interviews, which are designed for administration by lay interviewers, identified more patients with low‐level depressive symptoms as depressed but fewer patients with high‐level symptoms. These results are consistent with the idea that semi‐structured interviews most closely replicate clinical interviews done by trained professionals. Fully structured interviews are less resource‐intensive options because they are completely scripted and allow for minimal or no judgment, since they are designed to be administered by research staff without diagnostic skills. They may, however, misclassify major depression in substantial numbers of patients. In the EPDS IPDMA database, the SCID was the most common semi‐structured interview. In a sensitivity analysis, we included two additional studies from the database that used semi‐structured interviews other than the SCID.

### 2.2 Data sources, search strategy, and study selection

A medical librarian searched Medline, Medline In‐Process & Other Non‐Indexed Citations and PsycINFO via OvidSP, and the Web of Science Core Collection via ISI Web of Knowledge from inception to June 10, 2016, using a peer‐reviewed search strategy (McGowan et al., 2016) (see Supplementary material, Methods S2). We also reviewed reference lists of relevant reviews and queried contributing authors to attempt to identify non‐published studies. Search results were uploaded into RefWorks (RefWorks‐COS). After de‐duplication, remaining citations were uploaded into DistillerSR (Evidence Partners) for processing review results.

Two investigators independently reviewed titles and abstracts for eligibility. If either deemed a study potentially eligible, full‐text review was done by two investigators, independently, with disagreements resolved by consensus, consulting a third investigator when necessary. Translators were consulted for languages other than those for which team members were fluent.

### 2.3 Data contribution and synthesis

Authors of eligible datasets were invited to contribute de‐identified primary data, including EPDS scores and major depression classification status. We emailed corresponding authors of eligible primary studies at least three times, as necessary, with at least 2 weeks between each email. If we did not receive a response, we emailed co‐authors and attempted to contact corresponding authors by phone.

Prior to integrating individual datasets into our synthesized dataset, we compared published participant characteristics and diagnostic accuracy results with results from raw datasets and resolved any discrepancies in consultation with the original investigators. The number of participants and the number of cases from a primary study in the IPDMA dataset differed from the originally published primary study reports for some studies. There are several reasons for this. First, in some primary studies, some, but not all, participants met the inclusion criteria for the main IPDMA. For instance, we required administration of the EPDS index test and reference standard to be within a 2‐week period and only included participants aged 18 or older recruited from non‐psychiatric settings. We only included data from participants in primary studies who met these criteria. Second, the reference standard diagnostic category for the main IPDMA differed from that used in some published reports of primary studies. Some primary studies reported accuracy results for depression diagnoses broader than major depression, such as “major + minor depression” or “any depressive disorder”. We restricted our depression variable to major depression classification. Third, as part of our data verification process, we compared published participant characteristics and diagnostic accuracy results with results obtained using the raw datasets. When primary data that we received from investigators and original publications were discrepant, we identified and corrected errors in consultation with the original primary study investigators.

When primary datasets included statistical weights to reflect sampling procedures, we used the weights provided. For studies where sampling procedures merited weighting, but the original study did not weight, we constructed weights using inverse selection probabilities. This occurred, for instance, when all participants with positive screens and a random subset of participants with negative screens were administered a diagnostic interview.

### 2.4 Statistical analyses

First, for each primary study, we estimated three values: (1) the percentage of participants classified as having major depression based on the SCID, (2) the percentage of participants who scored above the cutoff threshold for all possible EPDS cutoffs (≥0 through ≥ 30), and (3) the difference of these percentages. Then, across all studies, we pooled prevalence for each EPDS cutoff, prevalence for the SCID, and the difference in prevalence from each study.

Second, we identified the EPDS cutoff with the smallest pooled difference. Then, for each included study, in addition to already estimated difference in prevalence based on the cutoff versus SCID major depression, we also estimated the ratio of prevalence based on the cutoff to that of the SCID. We plotted study‐level differences by sample size and determined the mean and median absolute difference and the range of differences across all studies. To illustrate the range of difference values that would be expected if a new study were to compare prevalence based on the prevalence match scoring approach to prevalence based on SCID major depression, we estimated a 95% prediction interval for the difference.

All meta‐analyses incorporated sampling weights and were conducted in R (R version R 3.6.0 and R Studio version 1.1.453) using the lme4 package (Bates, Mächler, Bolker, & Walker, 2015). To estimate pooled prevalence values, generalized linear mixed‐effects models with a logit link function were fit using the glmer function. To estimate pooled difference values, linear mixed‐effects models were fit using the lmer function. To account for correlation between subjects within the same primary study, random intercepts were fit for each primary study. To quantify heterogeneity, for each analysis, we calculated *τ* ^2^, which is the estimate of between‐study variance, and *I* ^2^, which quantifies the proportion of total variability due to the between‐study heterogeneity.

We conducted two sets of post‐hoc analyses. First, we repeated the prevalence match analysis excluding studies with SCID‐based prevalence >20% and >15%, separately, in order to assess results without studies that reported very high prevalence and ensure that results were consistent when only studies with more typical prevalence were included. For each subset of studies, we (1) identified the EPDS cutoff with the smallest pooled difference and (2) estimated the 95% prediction interval for the difference. Second, we investigated whether differences in prevalence for the EPDS prevalence match scoring approach and SCID were associated with study and participant characteristics in order to attempt to explain the heterogeneity we found. To do this, we fit additional linear mixed‐effects models for pooled prevalence difference, including age, pregnant versus postpartum status, country human development index (“very high”, “high”, or “low‐medium”) (United Nation's Development Programme, 2020), and study sample size as fixed‐effect covariates.

## 3 RESULTS

### 3.1 Search results and inclusion of primary study datasets

**FIGURE 1:**
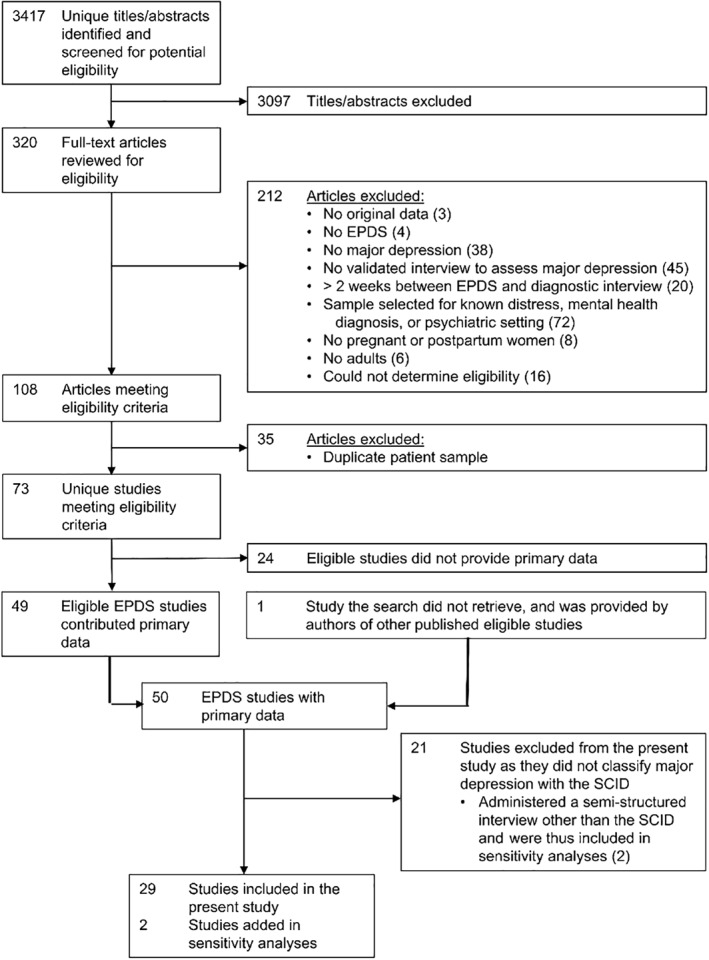
Flow diagram of the study selection process. CI, confidence interval; EPDS, Edinburgh Postnatal Depression Scale; SCID, Structured Clinical Interview for DSM

For the main IPDMA, of the 3417 unique titles and abstracts identified from the search, 3097 were excluded after title and abstract review and 212 after full‐text review. The 108 remaining articles comprised data from 73 unique samples, of which 49 (67%) contributed individual participant data. One additional study, which was subsequently published, was provided by the authors of an included study, for a total of 50 datasets. For our main analyses, we excluded 21 studies that classified major depression using a diagnostic interview other than the SCID, such that the sample for those analyses included 7315 participants (1017 major depression cases; prevalence 14%) from 29 primary studies (see Figure 1). Table 1 shows characteristics of each included study.

**TABLE 1:** Difference between EPDS ≥14 prevalence and SCID prevalence for each included study

Author, Year	Country	*N* Total	*N* (%) EPDS ≥ 14	*N* (%) Major depression	% Difference EPDS ≥ 14—Major Depression	Ratio: EPDS ≥ 14/Major depression
Aceti et al. (2012)^a^	Italy	44	16 (14.1%)	22 (19.4%)	−5.3%	0.7
Barnes, Senior, and MacPherson (2009)	UK	347	33 (9.5%)	25 (7.2%)	2.3%	1.3
Bavle et al. (2016)	India	318	13 (4.1%)	6 (1.9%)	2.2%	2.2
Beck and Gable (2001)	USA	150	15 (10.0%)	18 (12.0%)	−2.0%	0.8
Bunevicius, Kusminskas, Pop, Pedersen, and Bunevicius (2009)	Lithuania	230	11 (4.8%)	12 (5.2%)	−0.4%	0.9
Chaudron et al. (2010)	USA	187	39 (20.9%)	70 (37.4%)	−16.6%	0.6
de Figueiredo et al. (2015)^a^	Brazil	241	73 (20.8%)	94 (29.4%)	−8.6%	0.7
Garcia‐Esteve, Ascaso, Ojuel, and Navarro (2003)^a^	Spain	334	66 (6.9%)	36 (3.8%)	3.1%	1.8
Giardinelli et al. (2012)	Italy	588	39 (6.6%)	28 (4.8%)	1.9%	1.4
Helle et al. (2015)	Germany	224	29 (12.9%)	12 (5.4%)	7.6%	2.4
Hickey, Boyce, Ellwood, and Morris‐Yates (1997)^a^	Australia	72	16 (4.7%)	31 (9.1%)	−4.4%	0.5
Howard et al. (2018)^a^	UK	527	114 (8.4%)	130 (9.6%)	−1.2%	0.9
Leonardou et al. (2009)	Greece	81	10 (12.3%)	4 (4.9%)	7.4%	2.5
Navarro et al. (2007)^a^	Spain	401	108 (8.1%)	84 (8.1%)	0.0%	1
Phillips, Charles, Sharpe, and Matthey (2009)	Australia	158	46 (29.1%)	42 (26.6%)	2.5%	1.1
Prenoveau et al. (2013)^a^	UK	219	33 (9.7%)	20 (6.0%)	3.7%	1.6
Quispel, Schneider, Hoogendijk, Bonsel, and Lambregtse‐van den Berg (2015)	Netherlands	36	0 (0.0%)	0 (0.0%)	0.0%	Not applicable
Radoš, Tadinac, and Herman (2013)	Croatia	272	19 (7.0%)	10 (3.7%)	3.3%	1.9
Robertson‐Blackmore et al. (2013)	USA	358	62 (17.3%)	29 (8.1%)	9.2%	2.1
Rochat, Tomlinson, Newell, and Stein (2013)	South Africa	104	37 (35.6%)	50 (48.1%)	−12.5%	0.7
Siu, Leung, Ip, Hung, and O'hara (2012)	China	805	86 (10.7%)	126 (15.7%)	−5.0%	0.7
Stewart, Umar, Tomenson, and Creed (2013)^a^	Malawi	186	25 (5.3%)	34 (10.1%)	−4.8%	0.5
Tandon, Cluxton‐Keller, Leis, Le, and Perry (2012)	USA	89	20 (22.5%)	25 (28.1%)	−5.6%	0.8
Tendais, Costa, Conde, and Figueiredo (2014)^a^	Portugal	141	13 (4.9%)	18 (7.6%)	−2.7%	0.6
Töreki et al. (2013)	Hungary	219	3 (1.4%)	7 (3.2%)	−1.8%	0.4
Töreki et al. (2014)	Hungary	265	10 (3.8%)	8 (3.0%)	0.8%	1.2
Tran et al. (2011)	Vietnam	359	8 (2.2%)	52 (14.5%)	−12.3%	0.2
Turner et al. (2009)	Italy	54	4 (7.4%)	5 (9.3%)	−1.9%	0.8
Vega‐Dienstmaier, Mazzotti Suárez, and Campos Sánchez (2002)	Peru	306	75 (24.5%)	19 (6.2%)	18.3%	3.9

Studies from IPDMA that used other semi‐structured interviews and were included in sensitivity analysis						
Pawlby, Sharp, Hay, and O'Keane (2008)^b^	UK	190	17 (8.9%)	34 (17.9%)	−8.9%	0.5
Tissot, Favez, Frascarolo‐Moutinot, and Despland (2015)^c^	Switzerland	65	5 (7.7%)	4 (6.2%)	1.5%	1.2

Abbreviations: EPDS, Edinburgh Postnatal Depression Scale; IPDMA, individual participant data meta‐analysis; SCID, Structured Clinical Interview for DSM.

^a^ Sampling weights were applied. Counts are based on actual numbers whereas percentages are weighted.

^b^ Diagnostic interview: The Clinical Interview Schedule.

^c^ Diagnostic interview: The Diagnostic Interview for Genetic Studies.

In sensitivity analyses, we included data from two additional studies that used a semi‐structured diagnostic interview other than the SCID (*N* participants: 255; *N* major depression cases: 38; prevalence 15%). This resulted in inclusion of data from 31 primary studies (*N* participants: 7570; *N* major depression cases: 1055; prevalence 14%). See Table 1.

### 3.2 Depression prevalence based on EPDS cutoffs and the SCID

**FIGURE 2:**
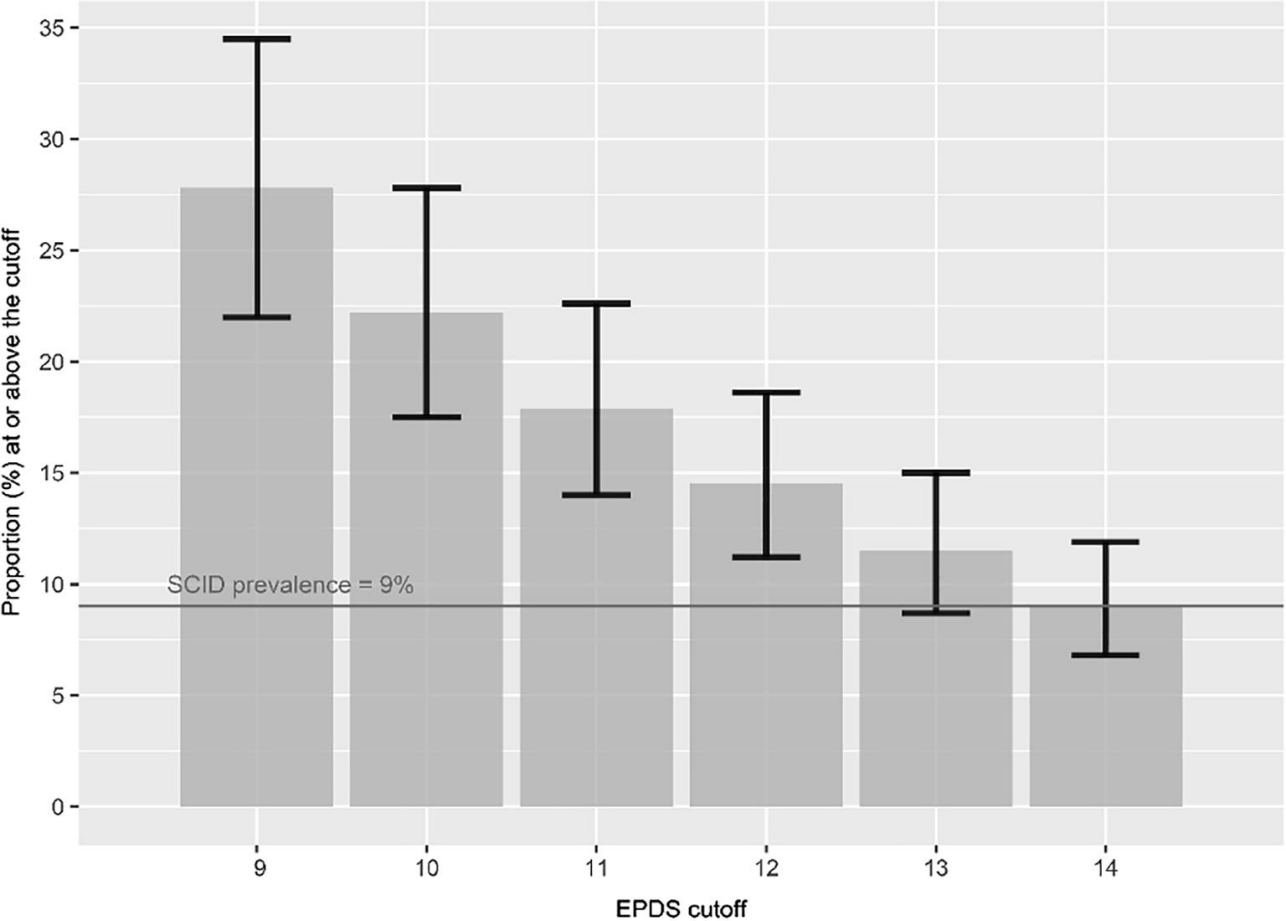
Prevalence estimates and 95% CI based on each EPDS cutoff threshold from **≥** 9 to **≥** 14. CI, confidence interval; EPDS, Edinburgh Postnatal Depression Scale; SCID, Structured Clinical Interview for DSM

Pooled prevalence estimates ranged from 27.8% (95% CI: 22.0%–34.5%, *τ* ^2^: 0.71, *I* ^2^: 96.5%) for EPDS cutoff ≥ 9 to 9.0% (95% CI: 6.8%–11.9%, *τ* ^2^: 0.66, *I* ^2^: 96.3%) for cutoff ≥ 14 (Figure 2, Table S2). The most commonly used cutoffs for estimating prevalence of ≥10 and ≥ 13 provided pooled prevalence estimates of 22.2% (95% CI: 17.5%–27.8%, *τ* ^2^: 0.64, *I* ^2^: 95.5%) and 11.5% (95% CI: 8.7%–15.0%, *τ* ^2^: 0.66, *I* ^2^: 96.4%), respectively. The pooled SCID major depression prevalence was 9.0% (95% CI: 6.5%–12.3%, *τ* ^2^: 0.87, *I* ^2^: 96.4%). See Figure 2.

### 3.3 Prevalence matching

**FIGURE 3:**
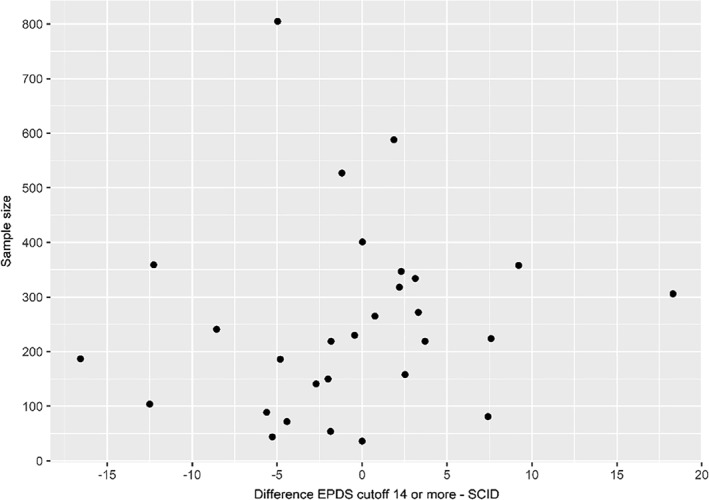
Dispersion of the differences between the prevalence estimates based on EPDS **≥** 14 and SCID major depression for each study, considering the study sample size. EPDS, Edinburgh Postnatal Depression Scale; SCID, Structured Clinical Interview for DSM

The pooled difference between the proportion of participants with EPDS ≥14 and SCID major depression prevalence across all studies was the smallest of all cutoff thresholds (−0.7%, 95% CI: −3.2% to 1.9%, *τ* ^2^: 0.004, *I* ^2^: 95.8%; Table S2). Across the 29 individual studies, however, differences ranged from −16.6% to 18.3% using that cutoff score (mean absolute difference: 5.1%; median absolute difference: 3.3%, Figure 3). Specifically, 20 (69%) studies using that cutoff were ≤0.75 times or ≥ 1.25 times the actual SCID‐based prevalence (Table 1). The 95% prediction interval for the difference between EPDS ≥14 and SCID‐based prevalence was −13.7% to 12.3%. Results were similar in the sensitivity analyses that included the two additional non‐SCID datasets (pooled EPDS ≥ 14 prevalence: 9.0%, 95% CI: 6.9%–11.7%, *τ* ^2^: 0.61, *I* ^2^: 96.5%; pooled major depression prevalence: 9.1%, 95% CI: 6.7%–12.3%, *τ* ^2^: 0.84, *I* ^2^: 96.6%; pooled difference: −0.9%, 95% CI: −3.4% to 1.6%; *τ* ^2^: 0.004, *I* ^2^: 96.0%; mean absolute difference: 5.1%; median absolute difference: 3.3%).

In post‐hoc analyses, for the 24 studies with SCID‐based prevalence ≤20%, prevalence based on the SCID was 6.8% (95% CI: 5.3%–8.5%, *τ* ^2^: 0.31, *I* ^2^: 95.1%). EPDS ≥14 was the closest match (pooled difference [95% CI]: 0.7% [−1.8% to 3.2%], *τ* ^2^: 0.003, *I* ^2^: 94.8%), and the 95% prediction interval for the difference was −10.8% to 12.2%. For the 22 studies with SCID‐based prevalence ≤15%, prevalence based on the SCID was 6.2% (95% CI: 5.0%–7.6%, *τ* ^2^: 0.21, *I* ^2^: 94.2%). EPDS ≥15 was the closest match (pooled difference [95% CI]: −0.2% [−2.3% to 1.9%], *τ* ^2^: 0.002, *I* ^2^: 93.6%), and the 95% prediction interval for the difference was −9.2% to 8.8%. Using data from all 29 included studies, no study or participant characteristics were significantly associated with differences in prevalence based on EPDS ≥14 versus SCID, with the exception of age, for which a one‐year increase in age was associated with 0.2% (95% CI: 0.1%–0.3%) decrease in “EPDS ≥ 14 minus SCID” prevalence.

## 4 DISCUSSION

The developers of the EPDS intended it to be a questionnaire that could detect symptoms of depression that are commonly experienced by women in the postpartum period but that would not be picked up by other scales (Cox et al., 1987). It was not intended to classify cases or estimate prevalence. Most studies that report the prevalence of depression in pregnancy or postpartum, however, are based on the proportion of women in the study who score above a cutoff threshold on a depression screening questionnaire, most commonly the EPDS. The EPDS cutoffs used to estimate prevalence in recently published studies ranged from ≥9 to ≥14, with ≥10 and ≥ 13 being the cutoffs most commonly used for this purpose. We found that, compared to SCID major depression prevalence, commonly used EPDS cutoffs overestimated prevalence. A cutoff of ≥10 on the EPDS generated a prevalence of 22%, and a cutoff of ≥13 generated prevalence of 11%, compared to 9% with the SCID.

Overall, the pooled prevalence based on an EPDS cutoff of ≥14 (9%) was closest to the pooled SCID major depression prevalence (9%). However, differences between prevalence based on the EPDS and SCID varied substantially across individual studies. The difference between EPDS‐ and SCID‐based prevalence ranged from −17% to 18%, and the estimated 95% prediction interval indicated that in the next study using both tools, the difference in prevalence could fall anywhere between −14% and 12%. Thus, although overall prevalence with EPDS ≥14 is similar to that of the SCID, if used to estimate prevalence in individual studies, it could considerably under or overestimate the true major depression prevalence in any given study. Differences between EPDS and SCID‐based estimates were not associated with sample size. We found that age was statistically significantly associated with the difference between EPDS ≥14 and SCID‐based prevalence, but a 1‐year difference in age was associated with only a 0.2% difference in prevalence; given the general similarity in ages of pregnant and postpartum women, this would not explain the large differences we found.

The results from this study are similar to findings from Levis et al. (2020) that compared prevalence based on the PHQ‐9 screening tool and the SCID. The most commonly used cutoff of PHQ‐9 ≥10 overestimated the SCID prevalence by approximately 12%. Prevalence matching for PHQ‐9 revealed that PHQ‐9 ≥14 provided a pooled prevalence estimate closest to SCID major depression prevalence. However, as in the present study, the difference in prevalence between PHQ‐9 ≥14 and the SCID varied considerably across individual studies.

It is common to report the proportion scoring at or above the cutoff threshold as prevalence of “depressive symptoms” or “clinically significant depressive symptoms” rather than suggesting that prevalence of depression has been reported. However, this does not resolve the problem. Diagnostic thresholds are designed to identify individuals with a condition or with a level of impairment that warrants attention, and there is no evidence that impairment from symptoms of depression becomes meaningful at or above these thresholds, which have been set for the purpose of screening, not for delineating impairment. Furthermore, while people with symptom scores above these thresholds have greater symptom impairment on average than those below the threshold, that would be the case for whatever threshold is set. Reporting percentages of women who score above different cutoffs may be useful for comparing levels of symptoms across samples, for instance. It should not, however, be characterized as “prevalence” or as a percentage of women who have “symptoms of depression” versus not having those symptoms.

This study was designed to evaluate how accurately the EPDS is for estimating prevalence; it did not evaluate the accuracy of the tool to identify individuals who may have depression and screen out those less likely to have depression. A strength of the present study is that it included data from 29 studies that fulfilled rigorous inclusion criteria and administered both the EPDS and the SCID. This made a direct comparison of prevalence estimates possible. A limitation is that the heterogeneity of the pooled prevalence based on both the SCID and EPDS was very high, despite well‐defined inclusion criteria and the narrow population of interest (women in pregnancy or postpartum). Furthermore, we compared prevalence based on two methods, but the estimation of the true depression prevalence in pregnancy and postpartum was out of the scope of this IPDMA, and the set of included primary studies may not be representative. Additionally, there were few studies with very large sample sizes (e.g., >400), and our examination of the association between sample size and differences between estimation methods may have been limited by this. Another was that the search included studies only through June 2016.

In conclusion, our findings show that EPDS is not able to accurately and reliably estimate depression prevalence in individual studies. Estimates based on the most commonly used cutoffs of ≥10 and ≥ 13 overestimate prevalence. Estimates based on a cutoff ≥14 were similar overall to SCID‐based estimates. However, there was variation between studies, and this cutoff could substantially under or overestimate prevalence in individual studies compared to prevalence based on a diagnostic interview. Thus, the proportion above a cutoff threshold on the EPDS should not be reported as prevalence of depression. Instead, validated diagnostic interviews, which are designed to classify case status based on standard diagnostic criteria, should be used for this purpose. Clinicians should be aware that studies that estimate prevalence based on standard cutoffs of ≥10 and ≥13 will tend to generate estimates that are higher than what they might expect to see in their practice.

## CONFLICT OF INTERESTS

All authors have completed the ICJME uniform disclosure form and declare: no support from any organization for the submitted work; *no financial relationships with any organizations that might have an interest in the submitted work in the previous three years with the following exceptions:* Dr. Tonelli declares that he has received a grant from Merck Canada, outside the submitted work. Dr. Vigod declares that she receives royalties from UpToDate, outside the submitted work. Dr. Beck declares that she receives royalties for her Postpartum Depression Screening Scale published by Western Psychological Services. Dr. Boyce declares that he receives grants and personal fees from Servier, grants from Lundbeck, and personal fees from AstraZeneca, all outside the submitted work. Dr. Howard declares that she has received personal fees from NICE Scientific Advice, outside the submitted work. No funder had any role in the design and conduct of the study; collection, management, analysis, and interpretation of the data; preparation, review, or approval of the manuscript; and decision to submit the manuscript for publication.

## AUTHOR CONTRIBUTIONS

Anita Lyubenova, Dipika Neupane, Brooke Levis, Yin Wu, Jill T. Boruff, John P. A. Ioannidis, Pim Cuijpers, Simon Gilbody, Lorie A. Kloda, Scott B. Patten, Ian Shrier, Roy C. Ziegelstein, Liane Comeau, Nicholas D. Mitchell, Marcello Tonelli, Simone N. Vigod, Andrea Benedetti, and Brett D. Thombs were responsible for the study conception and design. Jill T. Boruff and Lorie A. Kloda designed and conducted database searches to identify eligible studies. Franca Aceti, Jacqueline Barnes, Amar D. Bavle, Cheryl T. Beck, Carola Bindt, Philip M. Boyce, Andrea Bunevicius, Linda H. Chaudron, Nicolas Favez, Barbara Figueiredo, Lluïsa Garcia‐Esteve, Lisa Giardinelli, Nadine Helle, Louise M. Howard, Jane Kohlhoff, Laima Kusminskas, Zoltán Kozinszky, Lorenzo Lelli, Angeliki A. Leonardou, Valentina Meuti, Sandra N. Radoš, Purificación N. García, Susan J. Pawlby, Chantal Quispel, Emma Robertson‐Blackmore, Tamsen J. Rochat, Deborah J. Sharp, Bonnie W. M. Siu, Alan Stein, Robert C. Stewart, Meri Tadinac, S. Darius Tandon, Iva Tendais, Annamária Töreki, Anna Torres‐Giménez, Thach D. Tran, Kylee Trevillion, Katherine Turner, Johann M. Vega‐Dienstmaier were responsible for collection of primary data included in this study. Anita Lyubenova, Dipika Neupane, Brooke Levis, Ying Sun, Chen He, Ankur Krishnan, Parash M. Bhandari, Zelalem Negeri, Mahrukh Imran, Danielle B. Rice, Marleine Azar, Matthew J. Chiovitti, Nazanin Saadat, Kira E. Riehm, and Brett D. Thombs contributed to the title and abstract and full‐text review processes and data extraction for the meta‐analysis. Anita Lyubenova, Dipika Neupane, Brooke Levis, Yin Wu, Andrea Benedetti, and Brett D. Thombs contributed to the data analysis and interpretation. Anita Lyubenova, Dipika Neupane, Brooke Levis, Yin Wu, Andrea Benedetti, and Brett D. Thombs contributed to drafting the manuscript. All authors provided a critical review and approved the final manuscript. Andrea Benedetti and Brett D. Thombs are guarantors.

## Supporting information

Supplementary Material
