# Supplementary material for: Depression prevalence based on the Edinburgh Postnatal Depression Scale compared to Structured Clinical Interview for DSM DIsorders classification: Systematic review and individual participant data meta‐analysis
Source: Int J Methods Psychiatr Res. 2020 Oct 22;30(1):e1860. doi: 10.1002/mpr.1860 (PMC7992289; doi:10.1002/mpr.1860)
Supplement: Supplementary file 1 — Supplementary Material [file MPR-30-e1860-s001.docx]

**Supplementary material**

**eMethods 1**. Review of classification methods in studies reporting depression prevalence in perinatal settings

**eMethods 2**. Search strategies

**eTable 1.** Studies included in the review of classification methods in studies reporting depression prevalence in perinatal settings (N=53)

**eTable 2.** Pooled prevalence based on SCID major depression and based on all EPDS cutoff thresholds.

**eMethods 1**. Review of classification methods in studies reporting depression prevalence in perinatal settings

**OBJECTIVE**

The aim of this literature review was to identify what classification methods are used to classify depression in studies that report depression prevalence among pregnant and postpartum women, to determine how commonly the EPDS is used, and with what cutoff thresholds.

**METHODS**

**Search Strategy and Identification of Eligible Studies**

We included articles that were primary studies indicating in their title or abstract that they assessed prevalence of “depression”, “depressive disorders”, “major depression” or “major depressive disorder”. One of those terms had to be mentioned at least once in the title or in the abstract. Studies that reported prevalence of “depressive symptoms” were not included. We searched PubMed on May 24, 2019 for articles listed in PubMed from January 1, 2018 or later using the search term (((Depression[Title/Abstract] OR Depressive[Title/Abstract]) AND prevalence[Title/Abstract] AND (pregnan*[Title/Abstract] OR postpartum[Title/Abstract] OR post-partum[Title/Abstract] OR perinatal[Title/Abstract]))) AND ("2018"[Date - Create] : "3000"[Date - Create]). The search results were loaded in DistillerSR (Evidence Partners, Ottawa, Canada). Two investigators independently reviewed the titles and abstracts for eligibility. Conflicts were resolved by consensus and, if necessary, by involvement of a third investigator.

**Data Extraction from Eligible Studies**

One investigator extracted data into a form in DistillerSR; a second investigator reviewed the extracted data. Items that were extracted included whether a diagnostic interview or a self-report symptom questionnaire had been used to assess case status and prevalence, the name of the diagnostic interview or questionnaire, and the terminology used (e.g., “depression”, “depressive disorder”) to describe prevalence results in the article title and abstract. If the Edinburgh Postnatal Depression Scale (EPDS) was administered, the cutoff threshold used to classify cases was recorded. If any of this information could not be ascertained from the title or abstract, we reviewed the full-text version of the article.

**RESULTS**

The PubMed search identified 271 citations. Of these, 218 were excluded because they were either not primary studies or they did not report prevalence of “depression”, “depressive disorders”, “major depression” or “major depressive disorder.” From the remaining eligible 53 studies, 41 (77%) used questionnaires, 5 (9%) used validated diagnostic interviews, 1 (2%) used both and 6 (11%) used other methods (e.g., medical records). Among the 42 studies that reported prevalence based on a questionnaire, the most common questionnaire was the EPDS (26 studies; 62%). The second most frequently used questionnaire was the Beck Depression Inventory (4 studies; 10%). Of the 26 studies that used the EPDS, 9 (35%) used a cutoff threshold of ≥ 10, 8 (31%) used a cutoff of ≥ 13, 4 (15%) used a cutoff of ≥ 12, and one each (4% each) used cutoffs of ≥ 9 and ≥ 14. One study (4%) used both cutoffs of ≥ 10 and ≥ 13, and 2 studies (8%) did not report a cutoff.

**eMethods 2. Search strategies**

**MEDLINE (OvidSP)**

1. EPDS.af.

2. Edinburgh Postnatal Depression.af.

3. Edinburgh Depression Scale.af.

4. or/1-3

5. Mass Screening/

6. Psychiatric Status Rating Scales/

7. "Predictive Value of Tests"/

8. "Reproducibility of Results"/

9. exp "Sensitivity and Specificity"/

10. Psychometrics/

11. Prevalence/

12. Reference Values/

13. Reference Standards/

14. exp Diagnostic Errors/

15. Mental Disorders/di, pc [Diagnosis, Prevention & Control]

16. Mood Disorders/di, pc [Diagnosis, Prevention & Control]

17. Depressive Disorder/di, pc [Diagnosis, Prevention & Control]

18. Depressive Disorder, Major/di, pc [Diagnosis, Prevention & Control]

19. Depression, Postpartum/di, pc [Diagnosis, Prevention & Control]

20. Depression/di, pc [Diagnosis, Prevention & Control]

21. validation studies.pt.

22. comparative study.pt.

23. screen*.af.

24. prevalence.af.

25. predictive value*.af.

26. detect*.ti.

27. sensitiv*.ti.

28. valid*.ti.

29. revalid*.ti.

30. predict*.ti.

31. accura*.ti.

32. psychometric*.ti.

33. identif*.ti.

34. specificit*.ab.

35. cut?off*.ab.

36. cut* score*.ab.

37. cut?point*.ab.

38. threshold score*.ab.

39. reference standard*.ab.

40. reference test*.ab.

41. index test*.ab.

42. gold standard.ab.

43. or/5-42

44. 4 and 43

**PsycINFO (OvidSP)**

1. EPDS.af.

2. Edinburgh Postnatal Depression.af.

3. Edinburgh Depression Scale.af.

4. or/1-3

5. Diagnosis/

6. Medical Diagnosis/

7. Psychodiagnosis/

8. Misdiagnosis/

9. Screening/

10. Health Screening/

11. Screening Tests/

12. Prediction/

13. Cutting Scores/

14. Psychometrics/

15. Test Validity/

16. screen*.af.

17. predictive value*.af.

18. detect*.ti.

19. sensitiv*.ti.

20. valid*.ti.

21. revalid*.ti.

22. accura*.ti.

23. psychometric*.ti.

24. specificit*.ab.

25. cut?off*.ab.

26. cut* score*.ab.

27. cut?point*.ab.

28. threshold score*.ab.

29. reference standard*.ab.

30. reference test*.ab.

31. index test*.ab.

32. gold standard.ab.

33. or/5-32

34. 4 and 33

**Web of Science (Web of Knowledge)**

#1. TS=(EPDS OR “Edinburgh Postnatal Depression” OR “Edinburgh Depression Scale”)

#2. TS=(screen* OR prevalence OR “predictive value*” OR detect* OR sensitiv* OR valid* OR revalid* OR predict* OR accura* OR psychometric* OR identif* OR specificit* OR cutoff* OR “cut off*” OR “cut* score*” OR cutpoint* OR “cut point*” OR “threshold score*” OR “reference standard*” OR “reference test*” OR “index test*” OR “gold standard” OR “reliab*”)

#2 AND #1

*Databases=SCI-EXPANDED, SSCI, A&HCI*

**eTable 1.** Studies included in review of classification methods in studies reporting depression prevalence in perinatal settings (N=53)

| **PubMed ID** | **Author, Year** | **Interview or Questionnaire Used** | **EPDS Cutoff** |
| --- | --- | --- | --- |
| **Classification Tool: Questionnaires** | | | |
| 30863775 | Arambewela, Somasundaram, Jayasekara, and Kumbukage (2019) | BDI | Not Applicable |
| 30906594 | Belay, Moges, Hiksa, Arado, and Liben (2018) | BDI | Not Applicable |
| 29363374 | González-Mesa, Arroyo-González, Ibrahim-Díez, and Cazorla-Granados (2019) | BDI | Not Applicable |
| 29934032 | Horesh, Nukrian, and Bialik (2018) | BDI | Not Applicable |
| 29394914 | Zhou et al. (2018) | EPDS | 9 |
| 31048832 | Azad et al. (2019) | EPDS | 10 |
| 29298365 | Chagarlamudi, Kim, and Newton (2018) | EPDS | 10 |
| 30937313 | Chen, Wang, Ding, Shan, and Qi (2019) | EPDS | 10 |
| 30568841 | Chorwe-Sungani and Chipps (2018) | EPDS, MINI^a^ | 10 |
| 30767191 | Daoud et al. (2019) | EPDS | 10, 13 |
| 31060983 | Ding et al. (2019) | EPDS | 10 |
| 29702503 | Emerson, Mathews, and Struwe (2018) | EPDS | 10 |
| 30157909 | Kerie, Menberu, and Niguse (2018) | EPDS | 10 |
| 30579099 | Shwartz, Shoahm-Vardi, and Daoud (2019) | EPDS | 10 |
| 29412451 | Xiong, Deng, Wan, and Liu (2018) | EPDS | 10 |
| 30465367 | Ayyub, Sarfraz, Mir, and Salam (2018) | EPDS | 12 |
| 30320133 | Do, Nguyen, and Pham (2018) | EPDS | 12 |
| 31084823 | Nurbaeti, Deoisres, and Hengudomsub (2019) | EPDS | 12 |
| 29575943 | Peltzer, Rodriguez, Lee, and Jones (2018) | EPDS | 12 |
| 30924721 | Afshari, Tadayon, Abedi, and Yazdizadeh (2019) | EPDS | 13 |
| 29533871 | Badr, Ayvazian, Lameh, and Charafeddine (2018) | EPDS, BDI^b^ | 13 |
| 30374966 | Chan, Samaranayaka, and Paterson (2018) | EPDS | 13 |
| 30819195 | Duko, Ayano, and Bedaso (2019) | EPDS | 13 |
| 30627202 | Ezzeddin, Jahanihashemi, Zavoshy, and Noroozi (2018) | EPDS | 13 |
| 30258485 | Fantahun, Cherie, and Deribe (2018) | EPDS | 13 |
| 29890453 | González-Mesa et al. (2018) | EPDS | 13 |
| 30274879 | Vaezi, Soojoodi, Banihashemi, and Nojomi (2019) | EPDS | 13 |
| 29503587 | Alqahtani, Al Khedair, Al-Jeheiman, Al-Turki, and Al Qahtani (2018) | EPDS | 14 |
| 30704328 | Meky, Shaaban, Ahmed, and Mohammed (2019) | EPDS | Not Reported |
| 30927633 | Sharmin, Sarwar, Mumu, Taleb, and Flora (2019) | EPDS | Not Reported |
| 30947293 | Mokhele et al. (2019) | CES-D | Not Applicable |
| 30354901 | Soyannwo, Adebayo, and Sigbeku (2018) | Depression Anxiety and Stress Symptoms Scale | Not Applicable |
| 31086545 | Sabir, Nagi, and Kazmi (2019) | Goldberg’s depression scale | Not Applicable |
| 30263209 | Odinka et al. (2018) | HADS | Not Applicable |
| 29361916 | Gravensteen et al. (2018) | Hopkins Symptom Checklist | Not Applicable |
| 29760762 | Anokye, Acheampong, Budu-Ainooson, Obeng, and Akwasi (2018) | PHQ | Not Applicable |
| 30021539 | Larrabure-Torrealva et al. (2018) | PHQ | Not Applicable |
| 29776353 | Osok, Kigamwa, Stoep, Huang, and Kumar (2018) | PHQ | Not Applicable |
| 30051541 | Jiang et al. (2018) | Self-Rating Depression Scale | Not Applicable |
| 31077972 | Tang, Lu, Hu, and Zhong (2019) | Self-Rating Depression Scale | Not Applicable |
| 30092662 | Zhang et al. (2018) | Self-Rating Depression Scale | Not Applicable |
| 29854717 | Gebremichael, Yihune, Ajema, Haftu, and Gedamu (2018) | Self-Reported Questionnaire | Not Applicable |
| **Classification Tool: Interviews** | | | |
| 30568841 | Chorwe-Sungani and Chipps (2018) | EPDS, MINI^a^ | 10 |
| 30928189 | Lin et al. (2019) | MINI | Not Applicable |
| 31050785 | Rees et al. (2019) | MINI | Not Applicable |
| 30236801 | Reilly, Talcevska, Black, Matthey, and Austin (2019) | MINI | Not Applicable |
| 29306876 | Fellmeth et al. (2018) | SCID | Not Applicable |
| 29433610 | Howard et al. (2018) | SCID | Not Applicable |
| **Classification Tool: Other** | |  |  |
| 29557522 | Abdelaal, Mohamed, and Aly (2018) | Hospital Records | Not Applicable |
| 29417369 | Faherty, Matone, Passarella, and Lorch (2018) | Medical Records | Not Applicable |
| 29859378 | Nietola et al. (2018) | Medical Records | Not Applicable |
| 30314906 | Sherman and Ali (2018) | Medical Records | Not Applicable |
| 30734864 | Wallwiener et al. (2019) | Medical Records | Not Applicable |
| 30149388 | Lacy et al. (2018) | Medical Records, Medical History Questionnaire, Interview about psychiatric, developmental, medical, and family history | Not Applicable |

^a^MINI was administered to a subset of the sample and prevalence of depression based on both tools was reported

^b^BDI was administered only to the participants who scored above the cutoff threshold of EPDS and no prevalence estimate was based on it.

**Abbreviations**: BDI: Beck Depression Inventory; CES-D: Centre for Epidemiological Studies Depression Scale; EPDS: Edinburgh Postnatal Depression Scale; HADS: Hospital Anxiety and Depression Scale; PHQ: Patient Health Questionnaire; SCID: Structured Clinical Interview for DSM

**eTable 1 References**

Abdelaal, H., Mohamed, M. A., & Aly, H. (2018). Racial disparity, depression, and birth outcomes among pregnant teens. *Maternal and Child Health Journal*, *22*(10), 1400–1406. https://doi.org/10.1007/s10995-018-2519-9

Afshari, P., Tadayon, M., Abedi, P., & Yazdizadeh, S. (2019). Prevalence and related factors of postpartum depression among reproductive aged women in Ahvaz, Iran. *Health Care for Women International*, 1–11. https://doi.org/10.1080/07399332.2019.1578779

Alqahtani, A. H., Al Khedair, K., Al-Jeheiman, R., Al-Turki, H. A., & Al Qahtani, N. H. (2018). Anxiety and depression during pregnancy in women attending clinics in a University Hospital in Eastern province of Saudi Arabia: Prevalence and associated factors. *International Journal of Women's Health*, *10*, 101–108. https://doi.org/10.2147/IJWH.S153273

Anokye, R., Acheampong, E., Budu-Ainooson, A., Obeng, E. I., & Akwasi, A. G. (2018). Prevalence of postpartum depression and interventions utilized for its management. *Annals of General Psychiatry*, *17*, 18. https://doi.org/10.1186/s12991-018-0188-0

Arambewela, M. H., Somasundaram, N. P., Jayasekara, H. B. P. R., & Kumbukage, M. P. (2019). Prevalence of depression and associated factors among patients with Type 2 diabetes attending the diabetic clinic at a tertiary care hospital in Sri Lanka: A Descriptive Study. *Psychiatry Journal*, *2019*, 7468363. https://doi.org/10.1155/2019/7468363

Ayyub, H., Sarfraz, M., Mir, K., & Salam, F. T. (2018). Association of antenatal depression and household food insecurity among pregnant women: A Crosssectional Study From Slums Of Lahore. *Journal of Ayub Medical College, Abbottabad : JAMC*, *30*(3), 366–371.

Azad, R., Fahmi, R., Shrestha, S., Joshi, H., Hasan, M., Khan, A. N. S., . . . Billah, S. M. (2019). Prevalence and risk factors of postpartum depression within one year after birth in urban slums of Dhaka, Bangladesh. *PloS One*, *14*(5), e0215735. https://doi.org/10.1371/journal.pone.0215735

Badr, L. K., Ayvazian, N., Lameh, S., & Charafeddine, L. (2018). Is the effect of postpartum depression on mother-infant bonding universal? *Infant Behavior & Development*, *51*, 15–23. https://doi.org/10.1016/j.infbeh.2018.02.003

Belay, Y. A., Moges, N. A., Hiksa, F. F., Arado, K. K., & Liben, M. L. (2018). Prevalence of antenatal depression and associated factors among pregnant women attending antenatal care at Dubti hospital: A case of pastoralist region in Northeast Ethiopia. *Depression Research and Treatment*, *2018*, 1659089. https://doi.org/10.1155/2018/1659089

Chagarlamudi, H., Kim, J., & Newton, E. (2018). Associations of prepregnancy morbid obesity and prenatal depression with gestational weight gain. *Southern Medical Journal*, *111*(1), 23–29. https://doi.org/10.14423/SMJ.0000000000000756

Chan, J. E., Samaranayaka, A., & Paterson, H. (2018). Seasonal and gestational variation in perinatal depression in a prospective cohort in New Zealand. *The Australian & New Zealand Journal of Obstetrics & Gynaecology.* Advance online publication. https://doi.org/10.1111/ajo.12912

Chen, L., Wang, X., Ding, Q., Shan, N., & Qi, H. (2019). Development of postpartum depression in pregnant women with preeclampsia: A retrospective study. *BioMed Research International*, *2019*, 9601476. https://doi.org/10.1155/2019/9601476

Chorwe-Sungani, G., & Chipps, J. (2018). A cross-sectional study of depression among women attending antenatal clinics in Blantyre district, Malawi. *The South African Journal of Psychiatry*, *24*, 1181. https://doi.org/10.4102/sajpsychiatry.v24i0.1181

Daoud, N., O'Brien, K., O'Campo, P., Harney, S., Harney, E., Bebee, K., . . . Smylie, J. (2019). Postpartum depression prevalence and risk factors among Indigenous, non-Indigenous and immigrant women in Canada. *Canadian Journal of Public Health = Revue Canadienne De Sante Publique.* Advance online publication. https://doi.org/10.17269/s41997-019-00182-8

Ding, G., Niu, L., Vinturache, A., Zhang, J., Lu, M., Gao, Y., . . . Tian, Y. (2019). "Doing the month" and postpartum depression among Chinese women: A Shanghai prospective cohort study. *Women and Birth.* Advance online publication. https://doi.org/10.1016/j.wombi.2019.04.004

Do, T. K. L., Nguyen, T. T. H., & Pham, T. T. H. (2018). Postpartum depression and risk factors among Vietnamese women. *BioMed Research International*, *2018*, 4028913. https://doi.org/10.1155/2018/4028913

Duko, B., Ayano, G., & Bedaso, A. (2019). Depression among pregnant women and associated factors in Hawassa city, Ethiopia: An institution-based cross-sectional study. *Reproductive Health*, *16*(1), 25. https://doi.org/10.1186/s12978-019-0685-x

Emerson, M. R., Mathews, T. L., & Struwe, L. (2018). Postpartum depression screening for new mothers at Well Child Visits. *MCN. the American Journal of Maternal/Child Nursing*, *43*(3), 139–145. https://doi.org/10.1097/NMC.0000000000000426

Ezzeddin, N., Jahanihashemi, H., Zavoshy, R., & Noroozi, M. (2018). The prevalence of postpartum depression and its association with food insecurity among mothers referring to community health centers. *Iranian Journal of Psychiatry*, *13*(4), 280–287.

Faherty, L. J., Matone, M., Passarella, M., & Lorch, S. (2018). Mental health of mothers of infants with neonatal abstinence syndrome and prenatal opioid exposure. *Maternal and Child Health Journal*, *22*(6), 841–848. https://doi.org/10.1007/s10995-018-2457-6

Fantahun, A., Cherie, A., & Deribe, L. (2018). Prevalence and factors associated with postpartum depression among mothers attending public health centers of Addis Ababa, Ethiopia, 2016. *Clinical Practice and Epidemiology in Mental Health : CP & EMH*, *14*, 196–206. https://doi.org/10.2174/1745017901814010196

Fellmeth, G., Plugge, E. H., Carrara, V., Fazel, M., Oo, M. M., Phichitphadungtham, Y., . . . Mcgready, R. (2018). Migrant perinatal depression study: A prospective cohort study of perinatal depression on the Thai-Myanmar border. *BMJ Open*, *8*(1), e017129. https://doi.org/10.1136/bmjopen-2017-017129

Gebremichael, G., Yihune, M., Ajema, D., Haftu, D., & Gedamu, G. (2018). Perinatal depression and associated factors among mothers in Southern Ethiopia: Evidence from Arba Minch Zuria health and demographic surveillance site. *Psychiatry Journal*, *2018*, 7930684. https://doi.org/10.1155/2018/7930684

González-Mesa, E., Arroyo-González, M. L., Ibrahim-Díez, N., & Cazorla-Granados, O. (2019). Mood state at the beginning of the pregnancy and its influence on obstetric and perinatal outcomes. *Journal of Psychosomatic Obstetrics and Gynecology*, *40*(2), 106–113. https://doi.org/10.1080/0167482X.2018.1427726

González-Mesa, E., Kabukcuoglu, K., Körükcü, O., Blasco, M., Ibrahim, N., & Kavas, T. (2018). Cultural factors influencing antenatal depression: A cross-sectional study in a cohort of Turkish and Spanish women at the beginning of the pregnancy. *Journal of Affective Disorders*, *238*, 256–260. https://doi.org/10.1016/j.jad.2018.06.003

Gravensteen, I. K., Jacobsen, E.‑M., Sandset, P. M., Helgadottir, L. B., Rådestad, I., Sandvik, L., & Ekeberg, Ø. (2018). Anxiety, depression and relationship satisfaction in the pregnancy following stillbirth and after the birth of a live-born baby: A prospective study. *BMC Pregnancy and Childbirth*, *18*(1), 41. https://doi.org/10.1186/s12884-018-1666-8

Horesh, D., Nukrian, M., & Bialik, Y. (2018). To lose an unborn child: Post-traumatic stress disorder and major depressive disorder following pregnancy loss among Israeli women. *General Hospital Psychiatry*, *53*, 95–100. https://doi.org/10.1016/j.genhosppsych.2018.02.003

Howard, L. M., Ryan, E. G., Trevillion, K., Anderson, F., Bick, D., Bye, A., . . . Pickles, A. (2018). Accuracy of the Whooley questions and the Edinburgh Postnatal Depression Scale in identifying depression and other mental disorders in early pregnancy. *The British Journal of Psychiatry*, *212*(1), 50–56. https://doi.org/10.1192/bjp.2017.9

Jiang, W., Mo, M., Li, M., Wang, S., Muyiduli, X., Shao, B., . . . Yu, Y. (2018). The relationship of dietary diversity score with depression and anxiety among prenatal and post-partum women. *The Journal of Obstetrics and Gynaecology Research*, *44*(10), 1929–1936. https://doi.org/10.1111/jog.13728

Kerie, S., Menberu, M., & Niguse, W. (2018). Prevalence and associated factors of postpartum depression in Southwest, Ethiopia, 2017: A cross-sectional study. *BMC Research Notes*, *11*(1), 623. https://doi.org/10.1186/s13104-018-3730-x

Lacy, M., DeDios-Stern, S., Fredrickson, S., Parikh, S., Nader, T., & Frim, D. M. (2018). Prevalence of psychiatric diagnoses in pediatric chiari malformation Type 1. *Pediatric Neurosurgery*, *53*(6), 371–378. https://doi.org/10.1159/000488460

Larrabure-Torrealva, G. T., Martinez, S., Luque-Fernandez, M. A., Sanchez, S. E., Mascaro, P. A., Ingar, H., . . . Williams, M. A. (2018). Prevalence and risk factors of gestational diabetes mellitus: Findings from a universal screening feasibility program in Lima, Peru. *BMC Pregnancy and Childbirth*, *18*(1), 303. https://doi.org/10.1186/s12884-018-1904-0

Lin, P.‑Y., Chiu, T.‑H., Ho, M., Pei-Chen Chang, J., Hui-Chih Chang, C., & Su, K.‑P. (2019). Major depressive episodes during pregnancy and after childbirth: A prospective longitudinal study in Taiwan. *Journal of the Formosan Medical Association = Taiwan Yi Zhi.* Advance online publication. https://doi.org/10.1016/j.jfma.2019.03.003

Meky, H. K., Shaaban, M. M., Ahmed, M. R., & Mohammed, T. Y. (2019). Prevalence of postpartum depression regarding mode of delivery: A cross-sectional study. *The Journal of Maternal-Fetal and Neonatal Medicine*, 1–8. https://doi.org/10.1080/14767058.2019.1571572

Mokhele, I., Nattey, C., Jinga, N., Mongwenyana, C., Fox, M. P., & Onoya, D. (2019). Prevalence and predictors of postpartum depression by HIV status and timing of HIV diagnosis in Gauteng, South Africa. *PloS One*, *14*(4), e0214849. https://doi.org/10.1371/journal.pone.0214849

Nietola, M., Heiskala, A., Nordström, T., Miettunen, J., Korkeila, J., & Jääskeläinen, E. (2018). Clinical characteristics and outcomes of psychotic depression in the Northern Finland Birth Cohort 1966. *European Psychiatry: the Journal of the Association of European Psychiatrists*, *53*, 23–30. https://doi.org/10.1016/j.eurpsy.2018.05.003

Nurbaeti, I., Deoisres, W., & Hengudomsub, P. (2019). Association between psychosocial factors and postpartum depression in South Jakarta, Indonesia. *Sexual & Reproductive Healthcare*, *20*, 72–76. https://doi.org/10.1016/j.srhc.2019.02.004

Odinka, J. I., Nwoke, M., Chukwuorji, J. C., Egbuagu, K., Mefoh, P., Odinka, P. C., . . . Muomah, R. C. (2018). Post-partum depression, anxiety and marital satisfaction: A perspective from Southeastern Nigeria. *The South African Journal of Psychiatry*, *24*, 1109. https://doi.org/10.4102/sajpsychiatry.v24i0.1109

Osok, J., Kigamwa, P., Stoep, A. V., Huang, K.‑Y., & Kumar, M. (2018). Depression and its psychosocial risk factors in pregnant Kenyan adolescents: A cross-sectional study in a community health Centre of Nairobi. *BMC Psychiatry*, *18*(1), 136. https://doi.org/10.1186/s12888-018-1706-y

Peltzer, K., Rodriguez, V. J., Lee, T. K., & Jones, D. (2018). Prevalence of prenatal and postpartum depression and associated factors among HIV-infected women in public primary care in rural South Africa: A longitudinal study. *AIDS Care*, *30*(11), 1372–1379. https://doi.org/10.1080/09540121.2018.1455960

Rees, S. J., Fisher, J. R., Steel, Z., Mohsin, M., Nadar, N., Moussa, B., . . . Silove, D. (2019). Prevalence and risk factors of major depressive disorder among women at public antenatal clinics from refugee, conflict-affected, and Australian-born backgrounds. *JAMA Network Open*, *2*(5), e193442. https://doi.org/10.1001/jamanetworkopen.2019.3442

Reilly, N., Talcevska, K., Black, E., Matthey, S., & Austin, M.‑P. (2019). A comparison of the interviewer-administered phone and self-complete online versions of the computerized eMINI 6.0 in a sample of pregnant women. *Journal of Affective Disorders*, *242*, 265–269. https://doi.org/10.1016/j.jad.2018.08.055

Sabir, M., Nagi, M. L. F., & Kazmi, T. H. (2019). Prevalence of antenatal depression among women receiving antenatal care during last trimester of pregnancy in a tertiary care private institute of Lahore. *Pakistan Journal of Medical Sciences*, *35*(2), 527–531. https://doi.org/10.12669/pjms.35.2.649

Sharmin, K. N., Sarwar, N., Mumu, S. J., Taleb, A., & Flora, M. S. (2019). Postnatal depression and infant growth in an urban area of Bangladesh. *Midwifery*, *74*, 57–67. https://doi.org/10.1016/j.midw.2019.03.014

Sherman, L. J., & Ali, M. M. (2018). Diagnosis of postpartum depression and timing and types of treatment received differ for women with private and Medicaid coverage. *Women's Health Issues*, *28*(6), 524–529. https://doi.org/10.1016/j.whi.2018.08.007

Shwartz, N., Shoahm-Vardi, I., & Daoud, N. (2019). Postpartum depression among Arab and Jewish women in Israel: Ethnic inequalities and risk factors. *Midwifery*, *70*, 54–63. https://doi.org/10.1016/j.midw.2018.12.011

Soyannwo, T., Adebayo, A. M., & Sigbeku, O. (2018). Mental health problems of reproductive age group women in a rural community of south west Nigeria. *Journal of Mental Health*, 1–7. https://doi.org/10.1080/09638237.2018.1487533

Tang, X., Lu, Z., Hu, D., & Zhong, X. (2019). Influencing factors for prenatal stress, anxiety and depression in early pregnancy among women in Chongqing, China. *Journal of Affective Disorders*, *253*, 292–302. https://doi.org/10.1016/j.jad.2019.05.003

Vaezi, A., Soojoodi, F., Banihashemi, A. T., & Nojomi, M. (2019). The association between social support and postpartum depression in women: A cross sectional study. *Women and Birth*, *32*(2), e238-e242. https://doi.org/10.1016/j.wombi.2018.07.014

Wallwiener, S., Goetz, M., Lanfer, A., Gillessen, A., Suling, M., Feisst, M., . . . Wallwiener, M. (2019). Epidemiology of mental disorders during pregnancy and link to birth outcome: A large-scale retrospective observational database study including 38,000 pregnancies. *Archives of Gynecology and Obstetrics*, *299*(3), 755–763. https://doi.org/10.1007/s00404-019-05075-2

Xiong, R., Deng, A., Wan, B., & Liu, Y. (2018). Prevalence and factors associated with postpartum depression in women from single-child families. *International Journal of Gynaecology and Obstetrics*, *141*(2), 194–199. https://doi.org/10.1002/ijgo.12461

Zhang, Y., Muyiduli, X., Wang, S., Jiang, W., Wu, J., Li, M., . . . Yu, Y. (2018). Prevalence and relevant factors of anxiety and depression among pregnant women in a cohort study from south-east China. *Journal of Reproductive and Infant Psychology*, *36*(5), 519–529. https://doi.org/10.1080/02646838.2018.1492098

Zhou, C., Zheng, W., Yuan, Q., Zhang, B., Chen, H., Wang, W., . . . Yang, L. (2018). Associations between social capital and maternal depression: Results from a follow-up study in China. *BMC Pregnancy and Childbirth*, *18*(1), 45. https://doi.org/10.1186/s12884-018-1673-9

**eTable 2.** Pooled prevalence based on SCID major depression and based on all EPDS cutoff thresholds.

| **Classification Method** | **Pooled Prevalence (%)** | **95% CI of the Pooled Prevalence** | **Pooled Difference EPDS – SCID (%)** | **95% CI of the Pooled Difference** |
| --- | --- | --- | --- | --- |
| SCID Major Depression | 9.0 | 6.5, 12.3 | - | - |
| **EPDS Cutoff Threshold** | | | | |
| ≥ 0 | 86.1 | 80.9, 90.1 | 88.1 | 84.1, 92.1 |
| ≥ 1 | 93.9 | 90.6, 96.1 | 78.1 | 73.1, 83.1 |
| ≥ 2 | 86.1 | 80.9, 90.1 | 70.4 | 65.1, 75.6 |
| ≥ 3 | 77.0 | 69.7, 83.0 | 61.4 | 55.7, 67.1 |
| ≥ 4 | 67.0 | 59.2, 73.9 | 52.9 | 47.0, 58.7 |
| ≥ 5 | 58.0 | 50.2, 65.5 | 44.9 | 39.2, 50.5 |
| ≥ 6 | 49.4 | 41.8, 57.1 | 37.2 | 31.6, 42.7 |
| ≥ 7 | 40.8 | 33.6, 48.5 | 29.7 | 24.2, 35.2 |
| ≥ 8 | 33.8 | 27.1, 41.3 | 23.5 | 18.3, 28.7 |
| ≥ 9 | 27.8 | 22.0, 34.5 | 17.9 | 13.1, 22.7 |
| ≥ 10 | 22.2 | 17.5, 27.8 | 12.6 | 8.5, 16.7 |
| ≥ 11 | 17.9 | 14.0, 22.6 | 8.4 | 4.7, 12.0 |
| ≥ 12 | 14.5 | 11.2, 18.6 | 5.0 | 1.9, 8.2 |
| ≥ 13 | 11.5 | 8.7, 15.0 | 1.9 | -0.7, 4.6 |
| ≥ 14 | 9.0 | 6.8, 11.9 | -0.7 | -3.2, 1.9 |
| ≥ 15 | 7.5 | 5.6, 9.8 | -2.6 | -5.1, -0.1 |
| ≥ 16 | 5.9 | 4.4, 7.9 | -4.4 | -7.0, -1.8 |
| ≥ 17 | 4.7 | 3.4, 6.3 | -5.8 | -8.6, -3.0 |
| ≥ 18 | 3.5 | 2.6, 4.8 | -7.3 | -10.4, -4.3 |
| ≥ 19 | 2.5 | 1.9, 3.4 | -8.6 | -12, -5.2 |
| ≥ 20 | 1.9 | 1.4, 2.6 | -9.4 | -13.0, -5.9 |
| ≥ 21 | 1.4 | 1.0, 2.0 | -10.1 | -13.7, -6.4 |
| ≥ 22 | 1.0 | 0.7, 1.4 | -10.6 | -14.3, -6.9 |
| ≥ 23 | 0.6 | 0.4, 0.9 | -11.0 | -14.8, -7.2 |
| ≥ 24 | 0.4 | 0.4, 0.4 | -11.3 | -15.2, -7.4 |
| ≥ 25 | 0.2 | 0.1, 0.5 | -11.5 | -15.4, -7.5 |
| ≥ 26 | 0.2 | 0.1, 0.4 | -11.6 | -15.6, -7.7 |
| ≥ 27 | 0.1 | 0.0, 0.2 | -11.8 | -15.7, -7.8 |
| ≥ 28 | 0.0 | 0.0, 0.2 | -11.8 | -15.8, -7.9 |
| ≥ 29 | 0.0 | 0.0, 0.1 | -11.9 | -15.9, -7.9 |
| ≥ 30 | 0.0 | 0.0, 0.2 | -11.9 | -15.9, -7.9 |

**Abbreviations:** CI: confidence interval; EPDS: Edinburgh Postnatal Depression Scale; SCID: Structured Clinical Interview for DSM
